# Comparisons of the Effects of Low‐ or High‐Fat Diets Rich in Soybean Oil, Lard, and Tea Seed Oil on Markers of Blood–Brain Barrier and Neuroinflammation in Ovariectomized Mice

**DOI:** 10.1155/ijfo/4154633

**Published:** 2025-11-10

**Authors:** Shyh-Hsiang Lin, Cicilia Giofani Soetanto, Wan-Chun Chiu, Tzu-Wen L. Cross, Ning-Jo Kao, Yih-Ru Wu, Ching-I Lin, Poulami Jha

**Affiliations:** ^1^ School of Nutrition and Health Sciences, Taipei Medical University, Taipei, Taiwan, tmu.edu.tw; ^2^ School of Food Safety, Taipei Medical University, Taipei, Taiwan, tmu.edu.tw; ^3^ Department of Nutrition Science, Purdue University, West Lafayette, Indiana, USA, purdue.edu; ^4^ Department of Nutrition and Health Sciences, Kainan University, Taoyuan, Taiwan, knu.edu.tw; ^5^ Department of Neurology, Chang Gung Memorial Hospital, Linkou Medical Center, Taoyuan, Taiwan, cgmh.org.tw; ^6^ Department of Neurology, College of Medicine, Chang Gung University, Taoyuan, Taiwan, cgu.edu.tw; ^7^ Research Center for Food and Cosmetic Safety, College of Human Ecology, Chang Gung University of Science and Technology, Taoyuan, Taiwan, cgust.edu.tw; ^8^ Department of Nutrition and Health Sciences, Chang Gung University of Science and Technology, Taoyuan, Taiwan, cgust.edu.tw

**Keywords:** BDNF, blood–brain barrier, lard, neuroinflammation, soybean, tea seed oil

## Abstract

High‐fat diets (HFDs) may affect the blood–brain barrier (BBB) function and cause neuroinflammation. Different dietary oils may influence BBB and neuroinflammatory responses due to their unique fatty acid compositions. To elucidate the potential effects of different dietary oils, this study compared the effects of tea seed oil with those of soybean oil and lard on markers of BBB and neuroinflammation. Six‐week‐old ovariectomized mice were fed a normal or HFD for 12 weeks. The mice′s brain lipid profiles, brain‐derived neurotrophic factor (BDNF), BBB function–related markers (i.e., S100 calcium‐binding protein *β* [S100*β*], matrix metalloproteinase‐9 [MMP‐9], zonula occludens‐1, and glial fibrillary acidic protein [GFAP]), and inflammation marker levels were evaluated. When mice were fed diets containing large amounts of fat (i.e., HFDs), different types of fat seemed to elicit different effects on these measures. However, different dietary fats had no different effects on the measurements during normal diet intervention. The mice fed the tea seed oil–based HFD exhibited upregulated levels of BDNF and downregulated levels of GFAP, S100*β*, MMP‐9, and proinflammatory cytokines compared to those fed the soybean oil– and lard‐based HFDs. While HFDs might impact BBB function and neuroinflammation, the type of dietary fat consumed might play a significant role, suggesting that tea seed oil might have beneficial effects on BBB markers and neuroinflammation compared to soybean oil and lard in ovariectomized mice under HFD conditions. However, further studies are warranted to determine the effects of these HFDs on cellular composition within the brains of these ovariectomized mice.

## 1. Introduction

The types and amounts of fat consumed can significantly impact health outcomes. In the Western diet, high fat intake has been linked to an increased risk of metabolic diseases, including obesity [1, 2] as well as neurodegenerative disorders such as Alzheimer′s disease (AD) [3]. A HFD can increase the production of proinflammatory cytokines, including tumor necrosis factor alpha (TNF‐*α*), interleukin (IL)‐1*β*, and IL‐6, in the circulatory system, thereby activating the hypothalamic I*κ*B kinase *β* (IKK*β*)/nuclear factor kappa B (NF‐*κ*B) signaling pathway, resulting in neuroinflammation [4, 5]. Neuroinflammation also plays a role in the pathogenesis of neurodegenerative diseases (e.g., AD) [6].

Dietary fatty acid (FA) composition contributes to the regulation of neuroinflammatory responses. For example, dietary omega‐3 FAs can promote anti‐inflammatory responses in brain immune cells, whereas dietary saturated and omega‐6 FAs may trigger proinflammatory responses [7, 8], which are closely linked to neuroinflammation [6]. The BBB consists of several types of cells, including cerebral endothelial cells, astrocytes, and pericytes [9]. It acts as an important barrier in the central nervous system (CNS) and plays a key role in maintaining CNS homeostasis [9]. Astrocyte activation, marked by the overexpression of GFAP and S100*β*, can mediate inflammation and contribute to BBB disruption in neurodegenerative diseases (e.g., AD) [10–12]. Previous studies have shown that rodents consuming either a Western diet rich in saturated fat and sugar [13], or a HFD rich in saturated fat and cholesterol [14], developed hippocampal BBB impairment. The putative mechanisms linking HFDs to BBB disruption involve oxidative stress and inflammation in the brain, both of which are associated with astrocyte reactivity [15]. Increased oxidative stress and proinflammatory cytokine levels in the brain results in elevated MMP‐9 expression, contributing to BBB disruption and extracellular matrix damage [16]. Elevated MMP‐9 expression disrupts the endothelial basal lamina and impairs tight junction proteins, including claudin‐5, occludin, and zonula occludens‐1 (ZO‐1) [17]. The type of dietary fat is critical for BBB integrity. For instance, BBB disruption was observed in mice fed a HFD rich in saturated FAs, but not in mice fed a HFD rich in monounsaturated fatty acid (MUFA) or polyunsaturated fatty acid (PUFA) [18].

Evidence from a rodent study indicates that inflammation‐induced BBB disruption is more pronounced in males and older females than in young females, suggesting a potential protective role of estrogen in maintaining BBB function [19]. Estrogen has been shown to regulate BBB permeability [20], but in aged female animals, increased BBB permeability is observed, and estrogen treatment can worsen this condition [21]. Furthermore, aging is independently linked to increased BBB permeability, likely driven by neuroinflammatory changes associated with age [10]. The age‐related decline in estrogen levels, combined with BBB disruption, may increase the risk of cognitive decline and AD in older women [10, 20, 21].

Tea seed oil (*Camellia sinensis*), rich in oleic acid, is a potential alternative to olive oil [22]. The anti‐inflammatory properties of oleic acid have been implicated in inflammatory‐related diseases [23, 24]. A study using an AD mouse model demonstrated that dietary supplementation with extra‐virgin olive oil could alleviate BBB dysfunction and neuroinflammation [25]. However, the effects of daily consumption of tea seed oil on BBB function and neuroinflammation remain unknown. In this study, we investigated whether a tea seed oil–based normal diet or HFD would protect against BBB disruption and neuroinflammation in ovariectomized (OVX) Institute of Cancer Research (ICR) mice and compared the effects of tea seed oil–based normal and high‐fat diets with those of soybean oil– and lard‐based normal and high‐fat diets. An OVX female mouse model was employed in the present study to eliminate the variability caused by fluctuating ovarian hormones during the estrous cycle, which could influence brain responses to food intake [26]. Estrogen decline with aging is known to heighten the sensitivity of older females to the adverse effects of a HFD [27]. By inducing estrogen withdrawal in these mice, we aimed to minimize hormone‐related variability in the outcome measurements.

## 2. Materials and Methods

### 2.1. Animals and Experimental Procedures

Forty‐eight 6‐week‐old female ICR mice were obtained from the National Laboratory Animal Center (Taipei, Taiwan) and were housed under standard conditions (a 12‐h light/dark cycle, room temperature of 23^°^C ± 2^°^C, and relative humidity of 60*%* ± 10*%*). In the present study, ICR mice were selected because they are widely used in biomedical research, and previous studies have demonstrated their susceptibility to HFD‐induced obesity and metabolic disturbances [28, 29]. This is in line with the principle that rodent models should mimic relevant human disease features [30]. Each mouse underwent an OVX and was anesthetized with a Zoletil 50 (Virbac, France)/Rompun (Bayer, Germany) injection. All mice were housed under the aforementioned conditions for 1 week for recovery. The mice were then randomized into six groups: (1) soybean oil–based normal diet (N‐S, *n* = 8), (2) soybean oil–based HFD (HFD‐S, *n* = 8), (3) lard‐based normal diet (N‐L, *n* = 8), (4) lard‐based HFD (HFD‐L, *n* = 8), (5) tea seed oil–based normal diet (N‐T, *n* = 8), and (6) tea seed oil–based HFD (HFD‐T, *n* = 8). The determination of the sample size for each group was based on a previously established method, namely, the resource equation [31]. The mice consumed their assigned experimental diets for 12 weeks, and the body weight (BW) of each mouse was measured every week. Weekly weight gain (WG) was calculated for each mouse. At the end of the feeding period, all the mice were sacrificed via CO_2_ inhalation. Their brains were collected, immediately frozen in nitrogen, and maintained at −80°C until further analysis. The animal study protocol was approved by the Animal Ethics Committee of National Taiwan Sport University, Taoyuan, Taiwan (Protocol Code LAC‐515‐0407). All experiments were performed in accordance with relevant guidelines and regulations. The study was conducted in compliance with the ARRIVE guidelines.

### 2.2. Diets

The mice were fed either a normal diet based on the AIN‐93 formula or an HFD modified from the Harlan Teklad TD 93075 diet, as described by Zhuhua et al. [28] (Table 1). Both diets were prepared by mixing the listed ingredients in Table 1, placing them into metal containers, and weighing them daily. Food and water were provided ad libitum. In this study, the soybean‐based normal diet was treated as the control diet. To ensure a consistent comparison, the same amount of soybean oil was added to the lard‐ and tea seed oil–based HFDs. Water and food intake were recorded daily, and BW was monitored twice a week throughout the study period. For the HFD groups, food intake was restricted to match the caloric intake of the normal diet groups. The average daily water and food intake for each group was calculated at the end of the study, with food intake expressed in calories based on 3.8 kcal/g for normal diets and 5 kcal/g for high‐fat diets. In addition, the tea seed oil used in the N‐T and HFD‐T diets was analyzed by SGS Taiwan Co. Ltd. (Taipei, Taiwan) using gas chromatography. It was found to be rich in oleic acid (C18:1, n‐9; 77.88% of total FAs), followed by palmitic acid (C16:0; 9.07%) and linoleic acid (C18:2; n‐6, 8.79%), with smaller amounts of stearic acid (C18:0; 2.03%), linolenic acid (C18:3, n‐3; 0.32%), and other minor FAs (< 1.2%). The FA composition data of both soybean oil and lard were extracted from the Nutrient Composition Data Bank for Foods (NCDBF) provided by the Taiwan Food and Drug Administration [32], as both oils are commercially available and were purchased locally in Taiwan. According to the NCDBF, soybean oil contains 53.14% linoleic acid (C18:2, n‐6), 23.38% oleic acid (C18:1, n‐9), 10.9% palmitic acid (C16:0), 6% linolenic acid (C18:3, n‐3), and 4.11% stearic acid (C18:0). Lard contains approximately 36.7% oleic acid (C18:1, n‐9); 23% palmitic acid (C16:0), 19.5% linoleic acid (C18:2, n‐6), and 13.1% stearic acid (of C18:0).

**Table 1:** Animal diet composition (grams per kilogram) and energy provided by carbohydrates, proteins, and fat (percentage) in normal and high‐fat diets.

	**Diet (g/kg)**					
	**Normal diet** ^**a**^			**High-fat diet** ^**b**^		
	**Soybean oil**	**Lard**	**Tea seed oil**	**Soybean oil**	**Lard**	**Tea seed oil**
Cornstarch	465	465	465	245	245	245
Maltodextrin	155	155	155	80	80	80
Sucrose	100	100	100	100	100	100
Casein	140	140	140	200	200	200
L‐Cysteine	2	2	2	2	2	2
Soybean oil	40	0	0	275	40	40
Lard	0	40	0	0	235	0
Tea seed oil	0	0	40	0	0	235
Cellulose	50	50	50	50	50	50
Mineral mix (AIN‐93M‐MIX)	35	35	35	35	35	35
Vitamin mix (AIN‐93‐VX)	10	10	10	10	10	10
Choline bitartrate	3	3	3	3	3	3
Total	1000	1000	1000	1000	1000	1000
Calorie density (Cal/g)	3.8	3.8	3.8	5.0	5.0	5.0
**Energy composition**	**Normal diet** ^**a**^			**High-fat diet** ^**b**^		
Carbohydrates	76.3%			34.2%		
Proteins	14.5%			16.1%		
Fat	9.2%			49.7%		

^a^Modified from the AIN‐93 formula.

^b^Modified from the Harlan Teklad TD 93075 diet and Zhuhua et al. [28].

### 2.3. Western Blot Analysis

Samples of the whole brain tissues (50 mg) were homogenized in the RIPA buffer with a 1% protease inhibitor (PI) cocktail (PI + EDTA). The protein concentration was determined through a Bio‐Rad protein assay, with bovine serum albumin (Bionovas, Toronto, Canada) used as the standard. Equal amounts of protein were separated through 10% sodium dodecyl sulfate polyacrylamide gel electrophoresis (SDS‐PAGE) before being transferred to polyvinylidene difluoride membranes. The membranes were incubated with blocking buffer (5% skim milk in Tris‐buffered saline; pH 7.6) for 1 h at room temperature and were then incubated with specific primary antibodies overnight at 4°C. The primary antibodies were S100*β*, MMP‐9, TNF‐*α* (rabbit antibodies, 1:500; Proteintech Group, Rosemont, IL, United States), ZO‐1 (rabbit antibody, 1:250; Proteintech Group), IL‐6, NF‐*κ*B p65, V‐Rel avian reticuloendotheliosis viral oncogene homolog A (RELA), GFAP (rabbit antibodies, 1:1000; Proteintech Group), rabbit anti‐brain–derived neurotrophic factor (anti‐BDNF; 1:1000, Abcam, Cambridge, MA, United States), and mouse anti‐*β*‐actin (1:2000, Sigma, St. Louis, MO, United States). Anti‐rabbit or anti‐mouse immunoglobulin G (IgG; 1:5000, Sigma) was used as a secondary antibody. Immunoreactive proteins were detected using an enhanced chemiluminescence (ECL) detection system.

### 2.4. Gas Chromatography Analysis of FAs in Mouse Brain Tissues

The mouse whole brain tissue samples were homogenized in phosphate‐buffered saline, and lipids were extracted using the Folch method as described previously [33]. FAs were esterified by adding 1 mL of methanol and 500 *μ*L of boron trifluoride (BF_3_) to each sample, followed by heating in a water bath at 88°C for 1 h. After cooling, 1 mL of double‐distilled (dd)H_2_O and 2 mL of pentane were added to each sample. Next, each sample was centrifuged at 4°C and 3000 rpm for 10 min; thereafter, the supernatant was collected and dried with a vacuum pump. The samples were dissolved in hexane before the FA concentrations were measured.

A ThermoQuest gas chromatograph with a Restek Rtx‐2330 column (length: 30 m, inside diameter: 0.32 mm, film thickness: 0.32 *μ*m, cat. #10724; Bellefonte, PA, United States) was used to analyze the FA composition of each sample. The oven temperature was set to 90°C initially and was then increased to 250°C. The total analysis time, gasification temperature, split flow, and split ratio were 23 min, 260°C, 20 mL/min, and 10, respectively. Nitrogen was used as the mobile phase at a flow rate of 2 mL/min. A flame‐ionization detector with a base temperature of 260°C and an ignition threshold of 1 pA was used. The flow rates of air, hydrogen, and nitrogen were 350, 40, and 30 mL/min, respectively. The analysis was performed using the Chrom‐Card software. The concentration of each FA is expressed as the percentage of the content of that FA to the total FA content.

### 2.5. Statistical Analysis

Data are presented as mean ± standard error of *the* mean (SEM). A two‐way analysis of variance (ANOVA) was performed to assess the effects of diet (normal vs. HFD), type of dietary fat (soybean oil vs. lard vs. tea seed oil), and their interaction (diet × type of dietary fat) on brain FA composition and other measured markers. In parallel, an independent *t* test and one‐way ANOVA with a least significant difference (LSD) post hoc test were performed for multiple comparisons of the groups. Furthermore, a two‐way repeated measures ANOVA was performed for WG over the 12‐week period of the study. The BW was compared only between baseline and endpoint. Post hoc comparisons were conducted using LSD. The analyses were performed using IBM SPSS Statistics 19 (SPSS, New York, United States). A *p* value of < 0.05 was considered significant.

## 3. Results

### 3.1. Effects of the Diets on Mouse Water, Food and Energy Intakes, WG, and BW

Average daily water, food, and energy intakes across the study period are shown in Table 2. The two‐way ANOVA revealed significant main effects of both diet (*p* < 0.001, Table 2) and type of dietary fat (*p* < 0.001, Table 2), as well as a significant interaction between the two factors (*p* < 0.001, Table 2), for average daily water intake. For both average daily food and energy intakes, the main effect of diet (*p* < 0.001, Table 2) and the interaction between diet and type of dietary fat (*p* < 0.01, Table 2) was significant. The N‐S mice had significantly higher water intake than their counterparts in all other groups; subsequently, when mice were fed the diets based on the same type of fat, a significant difference in average daily water intake was only observed between the N‐S and HFD‐S groups (*p* < 0.05, Table 2). Regarding the average daily food and energy ingested per day, the mice in the HFD groups consumed significantly less food and energy/calories than those in the normal diet groups, regardless of the type of fat presented (*p* < 0.05, Table 2). On normal or high‐fat diets, there were no significant differences among the three different types of fat groups (*p* > 0.05, Table 2).

**Table 2:** Average daily water, food, and energy intakes of the OVX mice.

**Parameters**	**N-S**	**HFD-S**	**N-L**	**HFD-L**	**N-T**	**HFD-T**
Water intake (mL/day)	3.85 ± 0.17^c^	2.62 ± 0.05^a^	2.86 ± 0.11^ab^	2.60 ± 0.07^a^	2.95 ± 0.05^b^	3.11 ± 0.09^b^
Food intake (g/day)	4.41 ± 0.12^b^	2.92 ± 0.06^a^	4.77 ± 0.08^b^	2.88 ± 0.10^a^	4.65 ± 0.07^b^	2.91 ± 0.06^a^
Energy intake (Kcal/day)	16.75 ± 0.46^b^	14.61 ± 0.31^a^	18.13 ± 0.29^b^	14.38 ± 0.50^a^	17.69 ± 0.27^b^	14.53 ± 0.30^a^
**Two-way ANOVA results**						
**Source of variation**	**Diet (D)**		**Type of dietary diet (T)**		**D × T interaction**	
**Parameters**	**p** **value**		**p** **value**		**p** **value**	
Water intake	< 0.001		< 0.001		< 0.001	
Food intake	< 0.001		0.604		0.003	
Energy intake	< 0.001		0.881		0.005	

*Note:* Data presented as means ± SEM (*n* = 7 ~ 8). Different letter annotations within the same row indicate significant differences (*p* < 0.05) in one‐way ANOVA followed by LSD post hoc test. A two‐way ANOVA was also performed to determine the effects of diet, type of dietary fat, and their interaction (diet × type of dietary fat). For group abbreviations, see the Materials and Methods section.

**Figure 1:**
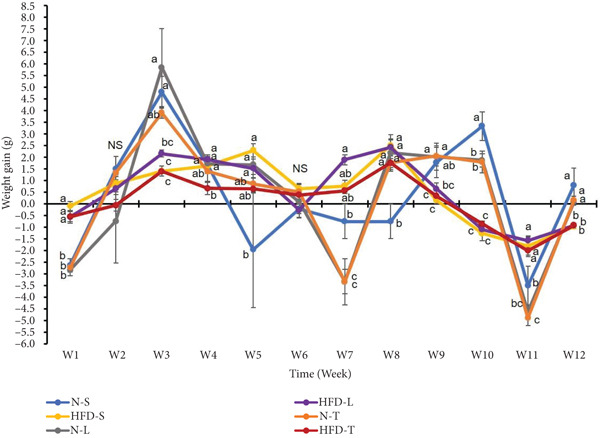
Effects of various diets on average weekly weight gain. Values are presented as means ± SEM (*n* = 8). Points not sharing the same letter(s) at each time (week) are statistically different (*p* < 0.05) from each other in two‐way repeated measures ANOVA followed by an LSD post hoc test. NS, not significant. For group abbreviations, see the Materials and Methods section.

As shown in Figure 1, it revealed that the main effect of “time‐point” and interactions between “time‐point” and “group” was significant for the WG (*p* < 0.001; Figure 1). This indicated that WG changed significantly over time in all groups, and the magnitude of these changes varied between groups. Regardless of type of dietary fat, HFD groups exhibited less fluctuation in WG over time compared with normal diet groups. Moreover, the post hoc test results showed that group differences in weekly WG were not consistent across time (Figure 1). Overall, under HFD conditions, the HFD‐T group tended to have lower average WG compared to its lard‐based counterparts (HFD‐L) throughout the study period, although these differences did not reach statistical significance (*p* > 0.05; Figure 1). It should be noted that the HFD‐T group also exhibited a more stable WG trajectory compared to the N‐T group. On the contrary, under normal diet conditions, the N‐T group had lower average WG than its soybean oil–based counterparts (N‐S) at several time points (Weeks 3, 7, 10, and 12), although these differences were not consistently significant (Figure 1). Regarding BW changes, the within group difference for average BW between endpoint and baseline was significant in all groups (*p* < 0.05), except for the HFD‐T group (*p* > 0.05; Table 3). Additionally, the average BW of mice in the HFD‐T group at the end of the study was significantly lower than their HFD‐S and HFD‐L counterparts (*p* < 0.05, Table 3). Taken together, these observations suggested that tea seed oil might exert a modest modulating effect on BW changes in both normal and high‐fat dietary conditions.

**Table 3:** Body weight (grams) of OVX mice at baseline and at the end of the study.

**BW (g)**	**N-S**	**HFD-S**	**N-L**	**HFD-L**	**N-T**	**HFD-T**
Baseline	34.96 ± 0.98^∗^	33.88 ± 0.77^∗^	34.98 ± 1.31^∗^	33.83 ± 0.69^∗^	34.73 ± 0.72^∗^	33.70 ± 0.80
Endpoint	39.00 ± 1.41^ab^	40.00 ± 1.67^a^	38.89 ± 1.25^ab^	40.59 ± 1.73^a^	37.55 ± 1.40^ab^	35.01 ± 1.13^b^

*Note:* Data expressed as mean ± SEM (*N* = 8). Asterisks ( ^∗^) within the same column and different letter annotations within the same row indicate significant differences (*p* < 0.05) in two‐way repeated measures ANOVA followed by LSD post hoc test. For brevity, only the data used for the comparison of the study baseline and endpoint are shown. For the average of body weight gain trajectories for each group, refer to Figure 1; for group abbreviations, see the Materials and Methods section.

### 3.2. Mouse Brain FA Compositions

The two‐way ANOVA revealed no significant main effects of diet and type of dietary fat for most brain FAs (*p* > 0.05, data not shown for brevity), except for C20:3 (n‐6). The main effect of diet for C20:3 (n‐6) was significant (*p* < 0.05, Table 4), while the interaction between diet and type of dietary fat for C18:2 (n‐6), C20:2 (n‐6), C20:3 (n‐6), and C22:6 (n‐3) was significant (*p* < 0.05, Table 4). Moreover, a post hoc LSD multiple comparison test suggested that the HFD‐S group had a significantly higher average concentration of linoleic acid (C18:2, n‐6) than the N‐S, HFD‐L, N‐T, and HFD‐T groups (*p* < 0.05; Table 4). The N‐L group had a significantly lower level of docosahexaenoic acid (DHA) (C22:6, n‐3) but higher levels of eicosadienoic acid (C20:2, n‐6) and dihomo‐*γ*‐linolenic acid (DGLA, C20:3, n‐6) than the other groups (*p* < 0.05; Table 4). The HFD‐L group had a significantly higher level of arachidonic acid (C20:4, n‐6) than the N‐L group (*p* < 0.05; Table 4).

**Table 4:** Mouse brain fatty acid composition (percentage).

	**N-S**	**HFD-S**	**N-L**	**HFD-L**	**N-T**	**HFD-T**	**p**	**p** **for interaction** ^**§**^
FAs								
Saturated FAs								
C14:0	0.53 ± 0.21	0.79 ± 0.08	0.60 ± 0.21	0.63 ± 0.18	0.59 ± 0.26	0.53 ± 0.13	0.93	0.7
C16:0	22.36 ± 1.11	25.15 ± 1.38	24.69 ± 0.63	23.17 ± 2.52	23.48 ± 1.00	24.26 ± 0.90	0.736	0.339
C17:0	0.26 ± 0.02	0.28 ± 0.02	0.20 ± 0.03	0.30 ± 0.04	0.24 ± 0.08	0.22 ± 0.05	0.585	0.362
C18:0	21.89 ± 0.65	21.84 ± 0.71	23.36 ± 0.37	22.96 ± 0.32	23.29 ± 0.65	22.14 ± 0.12	0.177	0.566
MUFAs								
C16:1	0.93 ± 0.17	0.62 ± 0.11	0.54 ± 0.06	0.58 ± 0.06	0.69 ± 0.17	0.71 ± 0.12	0.345	0.33
C18:1	22.09 ± 2.45	19.91 ± 3.45	17.11 ± 1.41	21.51 ± 1.42	21.46 ± 3.08	20.16 ± 0.47	0.687	0.331
PUFAs								
Omega‐3 PUFAs								
C18:3*α*	0.51 ± 0.11	0.40 ± 0.06	0.54 ± 0.17	0.73 ± 0.05	0.67 ± 0.24	0.60 ± 0.15	0.653	0.535
C20:5	0.27 ± 0.13	0.31 ± 0.14	0.14 ± 0.06	0.27 ± 0.14	0.30 ± 0.09	0.17 ± 0.03	0.812	0.493
C22:5	1.55 ± 0.85	0.90 ± 0.44	2.36 ± 0.29	0.40 ± 0.04^‡^	1.22 ± 1.02	1.13 ± 0.72	0.003	0.367
C22:6	16.47 ± 0.78^#^	13.76 ± 1.58^#^	10.35 ± 0.73	14.31 ± 0.28^‡^	13.02 ± 1.59^∗^	14.81 ± 0.27^#^	0.023	0.02
Omega‐6 PUFAs								
C18:2	0.65 ± 0.01	1.56 ± 0.28^∗^	1.30 ± 0.14	0.50 ± 0.03^†‡^	0.68 ± 0.46^†^	0.73 ± 0.14^†^	0.044	0.012
C18:3*γ*	0.08 ± 0.02	0.12 ± 0.09	0.02 ± 0.02	0.09 ± 0.02^‡^	0.07 ± 0.04	0.06 ± 0.02	0.031	0.634
C20:2	0.68 ± 019^#^	2.34 ± 1.66^#^	6.76 ± 0.87	0.88 ± 0.13^‡^	2.28 ± 1.99^#^	1.47 ± 0.46^#^	0.025	0.018
C20:3	0.40 ± 0.03^#^	0.51 ± 0.11^#^	1.07 ± 0.14	0.30 ± 0.03^‡^	0.49 ± 0.20^#^	0.39 ± 0.11^#^	0.008	0.009
C20:4	8.06 ± 0.77	7.48 ± 1.36	6.05 ± 0.46	8.38 ± 0.23^‡^	7.58 ± 0.92	8.49 ± 0.38	0.011	0.22
C22:4	3.27 ± 0.40	4.05 ± 0.52	5.86 ± 0.48	3.62 ± 0.61^‡^	3.95 ± 1.04	4.13 ± 0.67	0.044	0.088

*Note:* Data are expressed as means ± SEMs. Statistical analysis was performed using a two‐way ANOVA to determine the effects of diet, type of dietary fat, and their interaction (diet × type of dietary fat). In parallel, an independent *t* test was performed for the comparison of the means between groups fed the diets with the same type of dietary fat, and a one‐way ANOVA followed by an LSD post hoc test was performed for comparison of the means among all six groups. For group abbreviations, see the Materials and Methods section.

Abbreviations: FAs, fatty acids; MUFAs, monounsaturated fatty acids; PUFAs, polyunsaturated fatty acids.

^*^ *p* < 0.05 compared with the N‐S group. ^#^ *p* < 0.05 compared with the N‐L group. ^†^ *p* < 0.05 compared with the HFD‐S group in a one‐way ANOVA followed by an LSD post hoc test. ^‡^ *p* < 0.05 compared with the N‐L group in an independent *t* test. ^§^ *p* values for the interaction effects of diet and type of dietary fat are shown only (*p* < 0.05 indicates significance; see the Results section for details).

### 3.3. Effects of the Diets on S100*β*, MMP‐9, and ZO‐1 Levels

The HFD‐S and HFD‐L groups had significantly higher S100*β* levels than both the tea seed oil–based diet groups and higher MMP‐9 levels than any of the other groups (*p* < 0.05; Figure 2a,b). The type of dietary fat, rather than the amount of fat intake (normal vs. HFD), significantly affected S100*β* levels as indicated by a significant main effect of type of dietary fat (*p* < 0.01; Figure 2a). Furthermore, the two‐way ANOVA revealed significant main effects of diet and type of dietary fat for both MMP‐9 (*p* < 0.05 and *p* < 0.01; Figure 2b) and ZO‐1 (*p* < 0.05; Figure 2c) levels, whereas a significant interaction effect (diet × type of dietary fat) was observed only for MMP‐9 level (*p* < 0.05; Figure 2b). LSD post hoc comparisons revealed that the N‐T group had higher ZO‐1 levels than the other groups (*p* < 0.05), although the average ZO‐1 level of the N‐T group did not differ significantly from that of the N‐S group (Figure 2c).

Figure 2Effects of various diets on S100*β*, matrix metalloproteinase (MMP)‐9, and zonula occludens (ZO)‐1 levels in the brain. Upper panels: The representative western blot analysis for (a) S100*β*, (b) MMP‐9, and (c) ZO‐1 proteins in brain homogenates of N‐S, HFD‐S, N‐L, HFD‐L, N‐T, and HFD‐T treated mice. Each row of these blot images is a cropped image from one single blot data representing the expression of the target proteins and *β*‐actin. Middle panels: Levels of (a) S100*β*, (b) MMP‐9, and (c) ZO‐1 were defined as the ratio of each protein to the average *β*‐actin density. Lower panels: The tables show two‐way ANOVA results for (a) S100*β*, (b) MMP‐9, and (c) ZO‐1, including *p* values for the main effects of diet, type of dietary fat, and their interaction (diet × type of dietary fat).  ^∗^ *p* < 0.05 and  ^∗∗^ *p* < 0.01. Values are presented as means ± SEM (*n* = 4 or 5 biological replicates). Bars not sharing the same letter(s) are statistically different (*p* < 0.05) from each other in one‐way ANOVA followed by an LSD post hoc test. For group abbreviations, see the Materials and Methods section.

**Figure:**
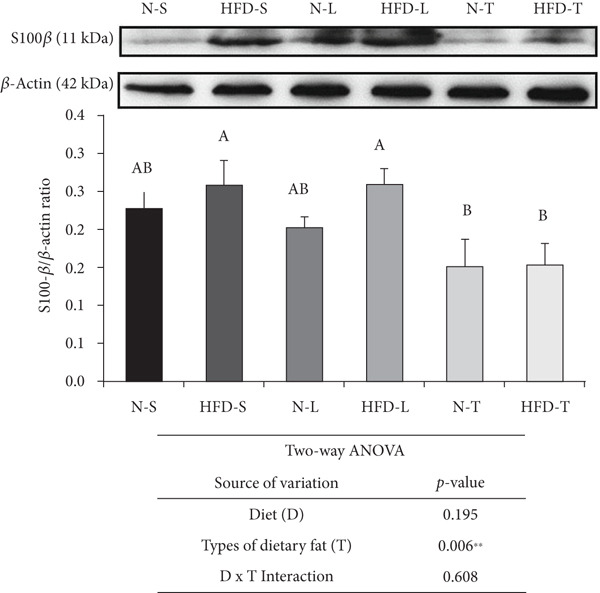
(a)

**Figure:**
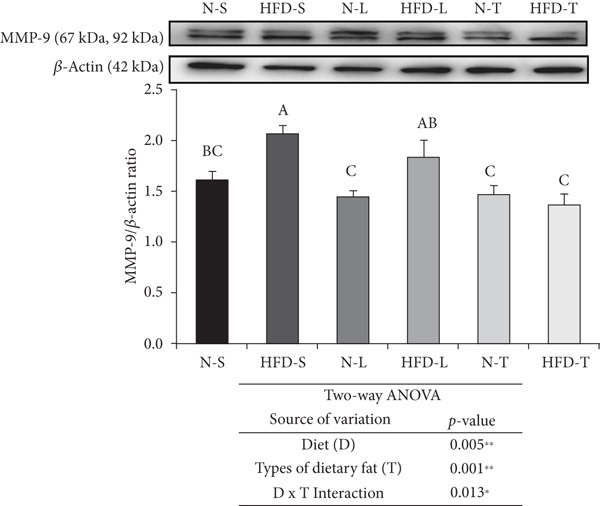
(b)

**Figure:**
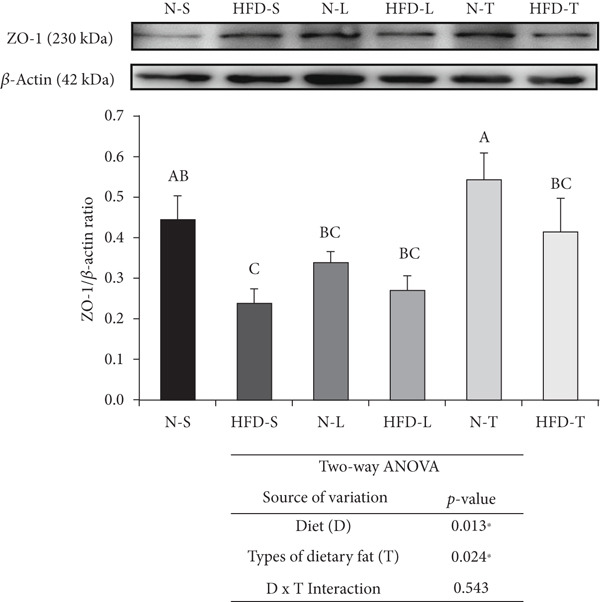
(c)

### 3.4. Effects of the Diets on GFAP Levels

As shown in Figure 3, the two‐way ANOVA revealed significant main effects of both diet (*p* < 0.05) and type of dietary fat (*p* < 0.05), suggesting that each factor independently influenced GFAP levels. Post hoc analysis using the LSD test showed that the HFD‐S and HFD‐L groups had significantly higher GFAP levels than both the tea seed oil–based diet groups (*p* < 0.05; Figure 3). No significant differences were observed between the tea seed oil–based diet groups and the N‐S and N‐L groups (*p* > 0.05; Figure 3), indicating that dietary fat type had a significant impact on GFAP levels.

Figure 3Effects of various diets on levels of glial fibrillary acidic protein (GFAP) in the brain. (a) The representative western blot analysis for GFAP protein in brain homogenates of N‐S, HFD‐S, N‐L, HFD‐L, N‐T, and HFD‐T treated mice. Each row of these blot images is a cropped image from one single blot data representing the expression of the target proteins and *β*‐actin. (b) GFAP levels were defined as the ratio of GFAP to the average *β*‐actin density. (c) The table shows the results of the two‐way ANOVA for GFAP, including *p* values for the main effects of diet, type of dietary fat, and their interaction (diet × type of dietary fat).  ^∗^ *p* < 0.05. Values are presented as means ± SEM (*n* = 4 or 5 biological replicates). Bars not sharing the same letter(s) are statistically different (*p* < 0.05) from each other in one‐way ANOVA followed by an LSD post hoc test. For group abbreviations, see the Materials and Methods section.

**Figure:**
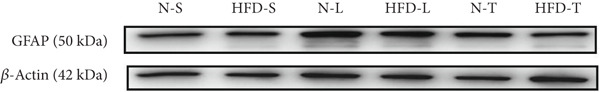
(a)

**Figure:**
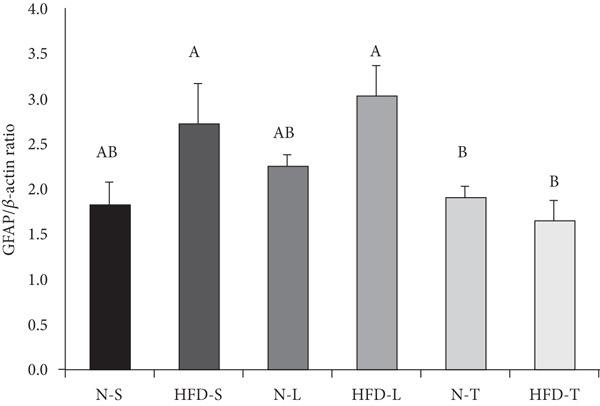
(b)

**Figure:**
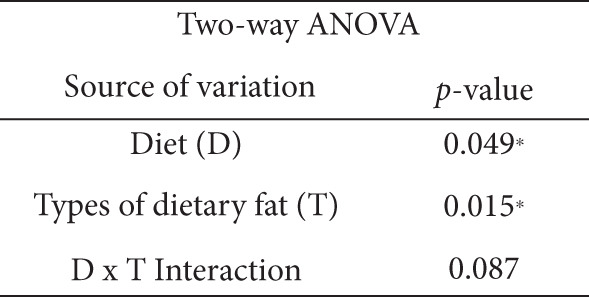
(c)

### 3.5. Effects of the Diets on NF‐*κ*B p65, TNF‐*α*, and IL‐6 Levels

As shown in Figure 4, the two‐way ANOVA revealed significant main effects of both diet (*p* < 0.05) and type of dietary fat (*p* < 0.001), as well as a significant interaction between the two factors (*p* < 0.05), for NF‐*κ*B p65. In contrast, none of these effects were significant for TNF‐*α* (*p* > 0.05). A significant main effect of diet was observed for IL‐6 (*p* < 0.05), indicating that IL‐6 levels may be influenced by the amount of fat intake (normal vs. HFD). Comparisons of multiple groups showed that the NF‐*κ*B p65 levels of the HFD‐S and HFD‐L groups were significantly higher than those of the other groups (*p* < 0.001; Figure 4a). The HFD‐T group had a significantly lower average NF‐*κ*B p65 level than the N‐L group (*p* < 0.001; Figure 4a). The HFD‐S and HFD‐L groups had higher TNF‐*α* and IL‐6 levels, although the difference was nonsignificant. The HFD‐T group had significantly lower TNF‐*α* levels than the HFD‐S and HFD‐L groups (*p* < 0.05; Figure 4b). The tea seed oil–based diet groups also had lower IL‐6 cytokine levels than the lard‐based diet groups (*p* < 0.05; Figure 4c).

Figure 4Effects of various diets on levels of nuclear factor (NF)‐*κ*B p65, tumor necrosis factor (TNF)‐*α*, and interleukin (IL)‐6 proteins in the brain. Upper panels: The representative western blot analysis for (a) NF‐*κ*B p65, (b) TNF‐*α*, and (c) IL‐6 proteins in brain homogenates of N‐S, HFD‐S, N‐L, HFD‐L, N‐T, and HFD‐T treated mice. Each row of these blot images is a cropped image from one single blot data representing the expression of the target proteins and *β*‐actin. Middle panels: Levels of (a) NF‐*κ*B p65, (b) TNF‐*α*, and (c) IL‐6 were defined as the ratio of each protein to the average *β*‐actin density. Lower panels: The tables show two‐way ANOVA results for (a) NF‐*κ*B p65, (b) TNF‐*α*, and (c) IL‐6, including *p* values for the main effects of diet, type of dietary fat, and their interaction (diet × type of dietary fat).  ^∗^ *p* < 0.05 and  ^∗∗∗^ *p* < 0.001. Values are presented as means ± SEMs (*n* = 4 or 5 biological replicates). Bars not sharing the same letter(s) are statistically different (*p* < 0.05) from each other in one‐way ANOVA followed by an LSD post hoc test. For group abbreviations, see the Materials and Methods section.

**Figure:**
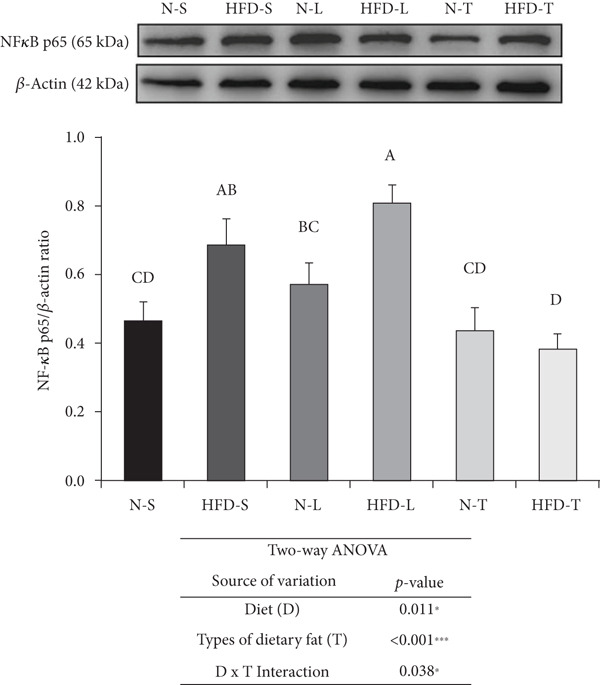
(a)

**Figure:**
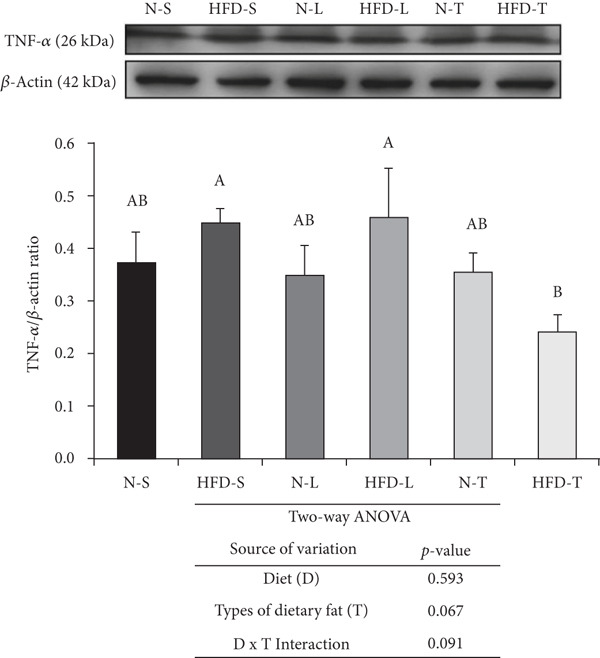
(b)

**Figure:**
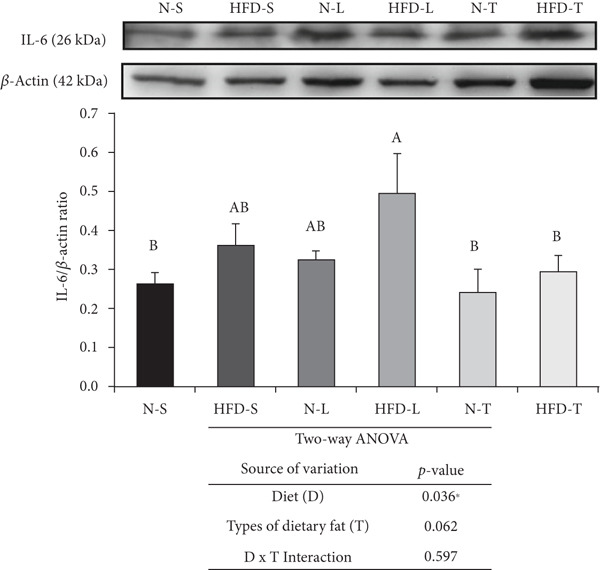
(c)

### 3.6. Effects of the Diets on BDNF Levels

In Figure 5, the main effect of type of dietary fat (*p* < 0.001), but not diet, and the interaction between diet and type of dietary fat were significant (*p* < 0.01). The amount of fat intake (normal vs. HFD) was not likely to affect BDNF levels. Furthermore, one‐way ANOVA followed by LSD post hoc tests showed that the HFD‐T group had a significantly higher average BDNF level than the other groups (*p* < 0.05; Figure 5), whereas the HFD‐L group had a lower average BDNF level than the soybean oil– and tea seed oil–based diet groups (*p* < 0.05; Figure 5).

Figure 5Effects of various diets on levels of brain‐derived neurotrophic factor (BDNF) in the brain. (a) The representative western blot analysis for BDNF protein in brain homogenates of N‐S, HFD‐S, N‐L, HFD‐L, N‐T, and HFD‐T treated mice. Each row of these blot images is a cropped image from one single blot data representing the expression of the target proteins and *β*‐actin. (b) BDNF levels were defined as the ratio of BDNF to the average *β*‐actin density. (c) The table shows the two‐way ANOVA results for BDNF, including *p* values for the main effects of diet, type of dietary fat, and their interaction (diet × type of dietary fat).  ^∗∗^ *p* < 0.01 and  ^∗∗∗^ *p* < 0.001. Values are presented as means ± SEMs (*n* = 5 or 6 biological replicates). Bars not sharing the same letter(s) are statistically different (*p* < 0.05) from each other in one‐way ANOVA followed by an LSD post hoc test. For group abbreviations, see the Materials and Methods section.

**Figure:**
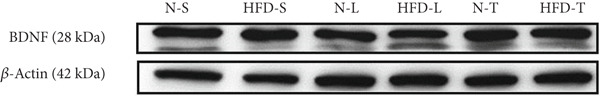
(a)

**Figure:**
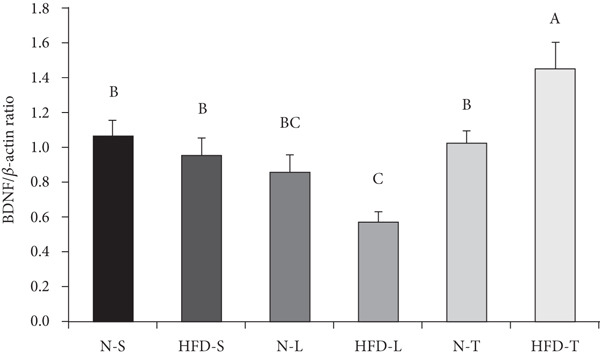
(b)

**Figure:**
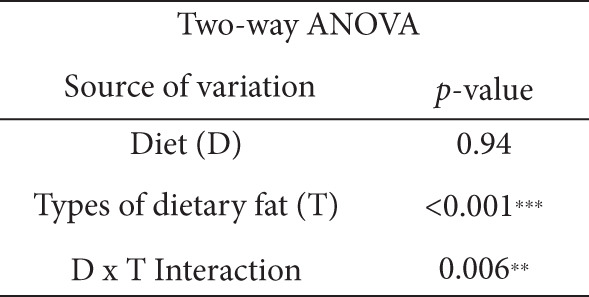
(c)

## 4. Discussion

The composition of dietary FA can indirectly influence brain functions related to mental health [34]. Although the Western diet and the Mediterranean diet have similar total fat content, their FA profiles differ significantly. The Western diet is characterized by high levels of saturated FAs and n‐6 PUFAs [35], whereas the Mediterranean diet is characterized by high levels of MUFAs and n‐3 PUFAs, primarily derived from olive and marine oils, respectively [36]. HFDs rich in saturated FAs and n‐6 PUFAs have been associated with cognitive impairment and depressive‐like behavior in rodent models [34]. By contrast, clinical evidence from a study suggested that adherence to a Mediterranean diet supplemented with olive oil improves cognitive function in older adults [37]. Tea seed oil, similar to olive oil, is rich in MUFAs while containing lower levels of saturated FAs and n‐6 PUFAs [22]. In the present study, the FA composition of the tea seed oil was comparable to that reported by Yang et al. [22], with oleic acid (C18:1, 75.78%–81.39%) as the predominant component, followed by linoleic acid (C18:2, 4.85%–10.79%), palmitic acid (C16:0, 7.68%–10.01%), and stearic acid (C18:0, 1.46%–2.97%). It may therefore serve as an alternative to olive oil and potentially exert similar health benefits.

Higher fat intake is associated with an increased prevalence of being overweight, obesity, and related metabolic diseases, primarily due to higher calorie consumption, which can result in excessive WG [2, 38]. However, the type of dietary fat may play a critical role in BW regulation, particularly in the context of HFD consumption. A previous study revealed that improving the quality of dietary fat was more effective than restricting total fat intake in terms of preventing obesity [39]. In the present study, the mice in the HFD‐T group exhibited a lower average BW compared to other HFD groups by the end of the study, suggesting that tea seed oil may contribute to BW regulation in the HFD‐treated OVX mice. The FA composition of tea seed oil likely underlies the observed BW changes. A previous study demonstrated that a MUFA‐rich diet with a high PUFA/saturated FA ratio may suppress WG and fat accumulation in obese hamsters [40]. Additionally, human studies suggest that a diet rich in MUFAs may prevent central body fat distribution, potentially through increased postprandial fat oxidation and enhanced diet‐induced thermogenesis [41, 42].

The brain PUFA profile may be affected by the types of dietary fat consumed over the previous 6–8 weeks [43]. Consistent with this notion, the present study observed that changes in the brain PUFA profile seemed to be more profound than those in the brain saturated FA or MUFA profile in response to varying dietary fat types and quantities. Additionally, previous evidence indicates that omega‐3 PUFAs play a crucial role in maintaining BBB integrity and mitigating neuroinflammation [44]. Similarly, we observed notable differences in brain DHA (C22:6, n‐3) among groups, which may have functional implications and warrant further investigation. Soybean oil, which is rich in omega‐6 linoleic acid [45], was associated with higher brain concentrations of linoleic acid in the HFD‐S group compared to other groups in the present study. Conversely, no significant between‐group differences were observed in brain saturated FA or MUFA levels, suggesting that dietary saturated FAs and MUFAs may have minimal impact on the brain FA profile. This may be because saturated FAs and MUFAs are produced de novo in the brain [46].

Astrocyte end‐feet play a crucial role in maintaining the function and integrity of the BBB by regulating the tight junction proteins, such as occludin and the claudins [47, 48]. Under pathological conditions, for example, neurodegenerative diseases, astrocyte activation, as reflected by increased GFAP [48] and S100*β* [11] levels, could lead to increased production of inflammatory mediators associated with BBB disruption [10]. In the present study, S100*β* and GFAP levels tended to increase in OVX mice fed the HFD‐S and HFD‐L compared to their normal diet–fed counterparts, although the difference did not reach statistical significance. Nonetheless, note that HFD feeding enabled astrocyte activation in a previous study [49], which is inconsistent with our findings. This discrepancy might be explained by the differences in caloric intake [49]. However, the HFD‐T group had significantly lower S100*β* and GFAP levels than the HFD‐S and HFD‐L groups. Consequently, astrocyte activation might be initiated in response to HFD‐S and HFD‐L, which might involve the early stages of BBB alteration. Plausibly, structural changes of the BBB might partially result from astrocyte activation, as astrocytes are key components of the BBB [50]. However, it should be noted that GFAP protein expression varies between brain regions and is observed in most astrocytes of mouse′s brains under physiological conditions [51]. Measuring GFAP protein expression from the whole brain lysates in this study was an initial approach to understanding HFD‐related biological pathways in the BBB of OVX mice. However, region‐specific insights remain unexplored, and potential contributions from neuronal or astrocytic injury to S100*β* levels cannot be excluded.

Astrocyte activation is also associated with increased MMP levels, which can degrade tight junction proteins and disrupt the BBB [52]. Specifically, MMP‐9 overexpression can contribute to BBB disruption via the proteolysis of the endothelial basal lamina and tight junction proteins (ZO‐1, claudin‐5, and occludin) [16, 17, 53, 54]. In the present study, the tight junction of the brain in the HFD‐S and HFD‐L groups, but not the HFD‐T group, may have been perturbed because they had higher MMP‐9 levels and lower ZO‐1 levels than those of their counterparts fed normal diets. Notably, soybean oil– and lard‐based HFD‐induced changes in MMP‐9 and ZO‐1 levels of OVX mice might be an early event of HFD‐related BBB disruption, while a tea seed oil–based HFD might limit such effects. HFDs have been reported to impair BBB integrity [55]; however, our study did not yield direct evidence to support this notion, primarily due to the limited assessment of tight junction proteins relevant to BBB integrity [56, 57].

BDNF plays crucial roles in neuron and glial cell development, synaptic plasticity, and BBB maintenance [56]. A previous study revealed that BBB disruption is accompanied by reduced BDNF levels and increased inflammatory markers at the transcript level after prolonged consumption of an HFD rich in lard [56]. Consistent with these findings, we observed that the mice in the HFD‐L had the lowest levels of BDNF, whereas those in the HFD‐T group had higher BDNF levels than those in other HFD groups. This aligns with earlier reports that lard‐based HFDs result in decreased BDNF levels [56, 58]. Intriguingly, our results suggest that tea seed oil, when incorporated into a HFD, might alleviate the adverse effects of the HFD itself on brain BDNF levels.

Moreover, regarding the effects of the oil type on neuroinflammatory responses, we observed elevated NF‐*κ*B p65, TNF‐*α*, and IL‐6 levels in the HFD‐S and HFD‐L groups, particularly compared to the HFD‐T group. By contrast, the NF‐*κ*B p65, TNF‐*α*, and IL‐6 levels of the mice fed normal diets showed similar levels of these markers regardless of the type of fat consumed. These findings suggest the anti‐inflammatory potential of tea seed oil in the context of HFDs, which have been reported to trigger neuroinflammation [55, 59]. Our data revealed that the mice in the HFD‐S and HFD‐L groups had higher levels of NF‐*κ*B p65 compared to those fed normal diets. Lard, which is high in palmitic acid, promotes NF‐*κ*B p65 expression, contributing to increased neuroinflammatory responses [24]. Similarly, linoleic acid, the predominant FA in soybean oil, has been shown to activate NF‐*κ*B in cultured endothelial cells, thereby contributing to cell dysfunction and inflammation [60]. Conversely, the low NF‐*κ*B p65 levels in HFD‐T treated OVX mice are indicative of the potential anti‐inflammatory effects of tea seed oil. This beneficial effect may be attributed, at least in part, to the FA composition of tea seed oil, which is rich in MUFAs [61], as well as its antioxidative components, including phenolic compounds and phytosterols [62, 63]. Previous studies have reported the regulatory role of dietary FAs in microglial cell–mediated neuroinflammation [61, 64]. Chronic consumption of a HFD rich in SFAs promotes a proinflammatory phenotype of microglia in the brain, particularly in conditions of obesity and metabolic syndrome, while an olive oil–based HFD rich in MUFAs activates the anti‐inflammatory phenotype of microglia [61]. However, the absence of microglial cell polarization analysis in our study represents a notable limitation, as our study primarily aimed to investigate the global impact on the entire brain in OVX mice in response to varying amounts and types of dietary fat. The specific effects of FA components on microglial cell responses were beyond the scope of this study. Interestingly, we observed no differences in TNF‐*α* and IL‐6 levels between the soybean oil– and lard‐based HFD groups and their normal diet–fed counterparts. Similar to a previous study, it was found that even diets with 41% of the calories from fat did not significantly affect TNF‐*α* or IL‐6 levels in the brain relative to the control group [65]. However, our results contrast with other studies reporting that chronic HFD feeding may induce a link between peripheral and neuroinflammation, as reflected by elevated TNF‐*α* or IL‐6 levels in both peripheral tissues and the hypothalamus [66, 67]. One possible explanation for these discrepancies might be differences in systemic inflammatory activation. However, blood samples were not taken in the present study, which prevented evaluation of systemic inflammation‐related markers.

Although this study has several limitations, it provides clear directions for future research. First, the assessment of BBB integrity was preliminary. Key tight junction proteins, such as claudin‐5 and occludin, were not examined, and more advanced methodologies, including immunofluorescence, image analysis, Evans Blue injections, and zymography, for direct inspection of BBB damage/leakage were beyond the scope of this study [56, 57]. Second, behavioral and cognitive assessments were not conducted, preventing us from establishing a direct link between observed molecular changes and potential functional outcomes in OVX mice′s brains. Third, the absence of microglial cell polarization analysis further limits our understanding of the cellular mechanisms underlying these effects. Last but not least, as this study was focused on the brain, peripheral metabolic and systemic inflammatory markers were not assessed. Consequently, the potential role of systemic inflammation in our observed findings remains unclear. Future research incorporating these functional, peripheral, and cellular analyses could provide a more comprehensive understanding of the mechanisms underlying HFD‐induced BBB alterations and their link to systemic inflammation.

## 5. Conclusions

This study revealed that different amounts and types of dietary fat had varying effects on BBB‐related markers, neuroinflammatory responses, and BDNF levels of OVX mice′s brains. Different types of fat did not significantly affect the markers of BBB function and neuroinflammation in OVX mice fed diets containing moderate amounts of fat. However, their effects became apparent to some extent in OVX mice fed diets containing large amounts of fat (i.e., HFDs). Specifically, our results imply that animal‐based fats such as lard and plant‐based oils like soybean oil in HFDs may alter the MMP‐9, ZO‐1, and NF‐*κ*B p65 levels in OVX mice′s brains. Of significance, tea seed oil, a plant‐based oil, appeared to mitigate these effects when used in HFDs. These findings suggest that tea seed oil has potential as a dietary intervention to alleviate the adverse effects of HFDs on BBB and neuroinflammatory markers and BDNF levels. Collectively, our findings suggest that consuming a moderate‐fat diet or replacing soybean oil or lard with tea seed oil in a HFD might have a reduced impact on the BBB‐related markers and neuroinflammatory responses in OVX mice′s brains; however, further studies are warranted to explore the effects of these dietary fats on cellular composition within the brains of OVX mice.

## Conflicts of Interest

The authors declare no conflicts of interest.

## Funding

This study was funded by the Chang Gung University of Science and Technology (ZRRPF3P0081).

## Data Availability

The data that support the findings of this study are available from the corresponding author (C‐I.L.) upon reasonable request.
